# Supplementary material for: Seroprevalence and SARS-CoV-2 invasion in general populations: A scoping review over the first year of the pandemic
Source: PLoS One. 2023 Apr 19;18(4):e0269104. doi: 10.1371/journal.pone.0269104 (PMC10118383; doi:10.1371/journal.pone.0269104)
Supplement: Attachment — Submitted filename: Response to reviewers.docx [file pone.0269104.s002.docx]

|  | **Journal Requirements:**  When submitting your revision, we need you to address these additional requirements. |
| --- | --- |
| **Requirement** | 1. Please ensure that your manuscript meets PLOS ONE's style requirements, including those for file naming. The PLOS ONE style templates can be found at  <https://journals.plos.org/plosone/s/file?id=wjVg/PLOSOne_formatting_sample_main_body.pdf> and  <https://journals.plos.org/plosone/s/file?id=ba62/PLOSOne_formatting_sample_title_authors_affiliations.pdf>. |
| **'author response'** | We have checked title and manuscript format requirements |
| **'author action'** | We have applied those requirements |
| **Requirement** | 2. Thank you for stating the following in the Acknowledgments Section of your manuscript:  [This research was funded by METROPOLE DU GRAND NANCY]  We note that you have provided funding information that is not currently declared in your Funding Statement. However, funding information should not appear in the Acknowledgments section or other areas of your manuscript. We will only publish funding information present in the Funding Statement section of the online submission form.  Please remove any funding-related text from the manuscript and let us know how you would like to update your Funding Statement. Currently, your Funding Statement reads as follows:   [Funding : The study was funded by Metropole du Grand (<https://www.grandnancy.eu/accueil/>). The funder had no role in study design, data collection and analysis, decision to publish, or preparation of the manuscript.]  Please include your amended statements within your cover letter; we will change the online submission form on your behalf. |
| **'author response'** | We updated and put the funding information in the Funding Statement section and removed any funding information from other places. |
| **'author action'** | The Funding Statement section now reads: “This research was funded by Metropole du Grand Nancy, CHRU de Nancy, and Inserm. The funders had no role in study design, data collection and analysis, decision to publish, or preparation of the manuscript.” |
| **Requirement** | 3. We note that you have stated that you will provide repository information for your data at acceptance. Should your manuscript be accepted for publication, we will hold it until you provide the relevant accession numbers or DOIs necessary to access your data. If you wish to make changes to your Data Availability statement, please describe these changes in your cover letter and we will update your Data Availability statement to reflect the information you provide. |
| **'author response'** | We did not mean to provide repository information for our data.We misunderstood the instructions to authors. Actually, all data are in the manuscript and supplementary files, and the data applicability statement is not applicable. |
| **'author action'** | Our Data availability statement has been changed to: “Not applicable” |
| **Requirement** | 4. Please include captions for your Supporting Information files at the end of your manuscript, and update any in-text citations to match accordingly. Please see our Supporting Information guidelines for more information: <http://journals.plos.org/plosone/s/supporting-information>. |
| **'author response'** | According to Reviewer #2’s suggestion, we have included the table into the manuscript instead of the supplement. The in-text citation and the supporting information file have been updated accordingly, and captions are provided at the end of the manuscript. |
| **'author action'** | The following captions are added at the end of the manuscript :  Figure 1: PRISMA Flowchart  Figure 2: Seroprevalence (%) in each continent over the observation period.  Table 1. Characteristics of country, methods, population of seroprevalence of the study. |
| **'additional author action'** | To better reflect the objective of this article, the title has been slightly amended into:  “Seroprevalence and SARS-CoV-2 invasion in general populations: a scoping review over the first year of the pandemic.”  Considering the important contribution of one author (TL), all other authors agreed to modify authors ranking. |
|  |  |
|  | **Reviewer #1** |
| **'reviewer comment'** | This was a review of SARS-CoV-2 seroprevalence between January 2020 and April 2021 that included 68 studies. While I applaud the authors for their efforts to narratively synthesis a heterogenous group of literature, and recognize the challenges of conducting evidence synthesis during the high-volume publication period of COVID-19, I have the following major concerns:  1) Given that this is a scoping review, the authors may need to review and adhere to the PRISMA scoping review guidelines. This template will help the authors to ensure the of their methods and reporting. |
| **'author response'** | We thank the reviewer for her/his appreciation and the advice. Actually, we used the PRISMA-Scr guidelines / checklist at first submission and this was submitted with the manuscript in the supporting information file. |
| **'author action'** | After revision, we have updated our PRISMA-Scr checklist, and attached it in the revised Supporting information file. |
|  |  |
| **'reviewer comment'** | 2) Scope and search. This was called a scoping review however, I'm not sure that it meets the methodological requirements of a scoping review. There were only 2 databases searched and the search strategy was quite rudimentary, with only a few terms. Scoping reviews are intended to cover as much of the available literature as possible, which typically involves searching multiple databases and the grey literature using a robust search strategy with involvement of a librarian. However, this search does not accomplish that. Was grey literature included? If not, why not? Was a health sciences librarian consulted about the search strategy? If not, why not? For this to be called a scoping review the search databases and search strategy need to be expanded and made more robust. |
| **'author response'** | We searched 2 databases for the whole period and searched medRXiv for the early months of 2021 only, on the premise that older paper would have been published, and papers in this database would have not been peer-reviewed. We realize this was insufficient. Moreover this was mentioned in a wrong place in our method section. We have now extended the medRXiv search to the whole period, i.e. cumulating 3 databases, mentioned it the method section and flow chart, and integrated the results. The key words for the search strategy was the final retained after several variations that did not bring more articles to our scrutiny. We did not include grey literature search, for we could not know how to search such literature in the particular context of this pandemic where so much suspicion was brought up for those non peer-reviewed publications. We could not afford, neither our institution, to involve a health sciences librarian. We managed to determine the search strategy collectively with all co-authors. To see whether we could make our search strategy more robust, we applied search strategies of other reviews (those we previously identified and those mentioned by this reviewer below) on our review period. As can be seen in the table below, the number of identified studies are largely diverse and though do not correlate with the number of studies retained by each author. Also, we can point that the reviews conducted by Chen, et al. or Bobrovitz, et al. did not target the same purpose than our (‘summarise serological surveys for SARS-COV-2 infections in humans’, and ‘seroprevalence surveys published in 2020’, respectively) i.e. all type of populations. By comparing more closely with Grant, et al. and with Rostami, et al., whose purpose were closer to our (‘population-based seroprevalence studies from Europe available as of 15 september 2020’ and ‘estimate the global and regional seroprevalence of SARS-COV-2 in people of the general population‘, respectively), we certainly missed some studies, namely 39 out of which 13 were relevant to our search, while we identified 9 relevant studies that they did not. Therefore, we reinforced our search strategies in two ways: 1) by looking more systematically at previous reviews, including their references, and 2) by searching medRXiv for the whole period, to incorporate relevant individual studies from these two sources. Table 1 : Search strategy from each author applied to our search period (period 1: Jan 1st, 2020 – April 10, 2021) and to more recent period (period 2: april 11th, 2021 – december 31st, 2021) Pubmed \| medRxiv \| Web of science; Bobrovitz \| \|; Period 1 : N=136 780 Period 2 : N=53 313 \| \| Period 1 : N=7 057 Period 2 : N=6 219; Chen \| \|; Period 1 : N=8 478 Period 2 : N=6 762 \| Period 1 : N=3 924 Period 2 : N=2 703 \| Period 1 : N=5 912 Period 2 : N=5 773; Grant \| \|; Period 1 : N=659 Period 2 : N=601 \| Period 1 : N=591 Period 2 : N=384 \|; Metzger \| \|; Period 1 : N=742 Period 2 : N=823 \| Period 1 : N=586 Period 2 : N=709 \| Period 1 : N=281 Period 2 : N=521; Rostami \| \|; Period 1 : N=11 126 (10 701+425) Period 2 : N=7 756 (7 480+276) \| \| |
| **'author action'** | The flow chart and method section have been amended accordingly. The reviews source have been identified. The 71 additional articles, including 6 reviews, were added to the search, analysed and results have been completed and amended accordingly. |
|  |  |
| **'reviewer comment'** | 3) Inclusion/exclusion criteria. Please could the authors define a key aspect of the inclusion criteria: what is a general population? |
| **'author response'** | This is an important question that may make a difference with other reviews, also explaining some heterogeneity in the articles selected. We have considered as general population a population-based sample, or a described general population sample, preferably (but not exclusively) obtained by random selection from a large population (survey or database). We have also included blood donors sample as proxy for general population, since they are likely not biased toward SARS-COV-2 seroprevalence, and we have presented them distinctly in the results. Exclusion criteria were health care workers, people attending a clinic or a hospital, and samples from a professional branch (industry, factory, farmers, university) and from a particular population (students, nursing home). To document this search we hand-searched all elicited articles instead of using the term “general population” in the key words, which proved to be too restrictive, as can be seen in the table2 below compared to table 1 above Table 2 : Search strategy from each author applied to our search period (Jan 1st, 2020 – April 10, 2021 ) adding « general population » in key-words Pubmed \| medRxiv \| Web of science; Bobrovitz \| \|; N=6 987 \| \| N=288; Chen \| \|; N=440 \| N=3 425 \| N=173; Grant \| \|; N=69 \| N=530 \|; Metzger \| \|; N=131 \| N=450 \| N=67; Rostami \| \|; N=616 (591 + 25) \| \| |
| **'author action'** | This is now made more explicit in the Method section. |
|  |  |
| **'reviewer comment'** | 4) Missing original studies and reviews. This is a fairly substantial issue with this article. The authors indicated that they included original studies and reviews. However, they are missing hundreds of original studies and some of the landmark systematic reviews on SARS-CoV-2 seroprevalence. This is demonstrated by an examination of the landmark reviews, which included many more original studies, despite being conducted at earlier points in the pandemic. All of these studies were available in peer-review or pre-print form prior to the end of the authors search date: A) Bobrovitz PLOS One (n=590 studies in the general population; 16(6): e0252617) (preprint available prior to end of authors search date) B) Chen in Lancet Global Health (n=84 studies in the general population; Volume 9, ISSUE 5, e598-e609, May 01, 2021) C) Rotami Clin Microbiol Infect (n=68 studies in the general population; 2021 Mar;27(3):331-340)  These landmark studies were also not discussed in the discussion section. Can the authors please explain why these reviews were not included or emphasized? And why the discrepancies in the number of original studies? To be a scoping review, you would expect that these studies would be included. |
| **'author response'** | We agree. This difference was mainly due to our definition of a general population.  We initially selected the Chen, et al. and the Grant, et al. reviews, but not the one by Bobrovitz, et al. nor the Rostami, et al. Those numbers of studies are not always those retained for their general population definition and would not be for our, as explained above.  We have now included 6 reviews in our search and analysis. |
| **'author action'** | The change in our search strategy has resulted in a large increase of the studies included (n=133) and the reviews (n=6) that helped identify additional studies. |
|  |  |
| **'reviewer comment'** | 5) Search date. Unfortunately, the literature on SARS-Cov-2 moves very quickly. I know it is very challenging to keep up to date with evidence synthesis and appreciate that limited resources and time play a role. However, this manuscript is already outdated. I know this is a difficult review comment to receive when doing evidence synthesis and for that I am sorry. But, according to the serotracker.com online database of SARS-CoV-2 - several hundred studies in the general population have been published. Given the significant literature published after the end of the search date, the authors would need to update their search to include some of these studies. Or at the very least explain how their snapshot from January 2020 - April 2021 provides added value in the context of the other larger and more inclusive reviews that have already been published. |
| **'author response'** | This point is important. It can be frequently the case when time passes with submission to previous journals that, moreover, did not take the effort to provide review of our manuscript. We appreciate the diplomatic way it is expressed, and the constructive review and criticism. Unfortunately, launching a new run of retrieving articles among hundreds of studies and extracting data seems a harder work that what we can do now, and unfortunately somewhat of an endless effort. As can be seen in table 1 above, the numbers of article to screen from the new period april to December 2021 would be about the same as what we already did, which is beyond our capacity.  More importantly, the scope of this review was purposedly focused on the first year of the pandemic to better understand the dynamic and factors for SARS-COV-2 penetration that could serve further as reference for setting up adapted measures to mitigate future epidemic (barriers, social distancing, evolutive vaccines according to molecular and biological monitoring of viruses, preventive or early treatments to avoid severity).  Besides, we have considerably enriched our search strategy and hence increased the number of relevant studies included (see 2) scope and search above). Our review points on the following:   - showing how the pandemic increasing trend of seroprevalence has developed in time and space over the firs year, - and documenting associated factors, in particular social factors exposing individuals to SARS-COV-2 infection. |
| **'author action'** | The scope and interest of focusing on the first year of the pandemic are better specified in the objective and the discussion, supported by a more comprehensive review of studies published over the period. |
|  |  |
| **'reviewer comment'** | 6) Results. The authors do not do draw a distinction when reporting results of reviews and original studies. These are different types of evidence and the reporting of their results should reflect this. |
| **'author response'** | Thank you for this advice. We have considered presenting the results separately. Actually the scope of this review was not to produce a synthesis of seroprevalence estimates or a meta analysis of risk factors for infection, but to identify and present the heterogeneity of estimates over time and space. Therefore we used the reviews and meta-analysis to identify missing studies, and we have presented the new full set of original studies only. However, we discuss the differences with previous reviews in the discussion. |
| **'author action'** | Results of all original studies are presented. Previous reviews are used to identify missing studies and discussed in the discussion. |
|  |  |
|  | **Reviewer #2** |
|  | Line numbers refer to those in the original manuscript submission |
| **'reviewer comment'** | This scoping review summarizes SARS-CoV-2 seroprevalence studies mainly covering the first/second pandemic waves internationally. This study is of high interest and could help to gain rapid and broad insights related to spread/dynamics from the early stages of the pandemic. However, there are some limitations and important (major) revisions need to be done. Some parts (see below) are kept very generic and are not structured well. While the study aims to summarize the seroprevalence progression over time and according to geographic locations, this is not clearly shown in the article (in general the specific aims and the mapping approach is not very clear). There is no clear strategy for the qualitative data synthesis and results should be written more clearly. Thus, it is hard to draw conclusion (e.g. it is written that the increase is exponential in the abstract, but this is not clearly reflected in the results. It’s generally not surprising that the prevalence increased over time. In my option it would be much more interesting to qualitatively describe the changes/locations in some more detail which has important implications. |
| **'author response'** | Thank you for the appreciation, these comments and helpful suggestions. |
| **'author action'** | We have reorganized the method section with more details and have tried to go more in depth in the qualitative description of the findings. |
|  |  |
| **'reviewer comment'** | Abstract: there is some information missing. Please provide some further information regarding eligibility (e.g all ages? Children as well?). The method is not stated. Was the data only qualitatively summarized, if so, how? Suggest not to abbreviate Web of Science. A conclusion is missing. |
| **'author response'** | We acknowledge the abstract was not structured and detailed enough. |
| **'author action'** | The abstract has been completed with requested informations |
|  |  |
| **'reviewer comment'** | Introduction: The aims are very broad. It should be stated that the study aims to summarize/map the studies according to time elapsed since pandemic outbreak, geographic region, summarizing risk factors etc. |
| **'author response'** | Thank you for your advice to focus more precisely on the review content. |
| **'author action'** | The aim of the study has been rewritten to better cover the work done. |
|  |  |
| **'reviewer comment'** | Method: in general, the structure of the methods section should be improved. Some aspects need clarification. For example, the authors start with a statement regarding databases that were searched and state further below that also preprints were searched (..to retrieve further articles from reference list screening). I suggest providing subtitles that contain eligibility, search strategy, data extraction. |
| **'author response'** | Thank you for the valuable advice |
| **'author action'** | The structure of the method section has been revisited according to suggestion and to PRISMA-Scr checklist. |
|  |  |
| **'reviewer comment'** | Please clarify eligibility criteria: which languages? Age range? Design of studies etc. |
| **'author response'** | These criteria are now clarified |
| **'author action'** | The method section has been completed accordingly |
|  |  |
| **'reviewer comment'** | Search strategy is unclear. Was the Search done only with key terms? Or MESH terms such as "Seroepidemiologic Studies"[Mesh]. The search strategy is not very broad – there is potential for missing studies. |
| **'author response'** | The search strategy was the final retained after several variations that did not bring more articles to our scrutiny. The use of MESH terms brought much less studies than key terms. |
| **'author action'** | It is now presented in a more clear fashion |
|  |  |
| **'reviewer comment'** | The selection process is not clearly stated. How was the stage 1 (title, abstract) and stage 2 done? Independently by two reviewers? |
| **'author response'** | This was done independently by two reviewers, then by a third one allowing discussion in case of discrepancy after abstract reading to obtain consensus for selection |
| **'author action'** | This is now clarified in the method section. |
|  |  |
| **'reviewer comment'** | Data extraction. The variables extracted are not well defined…what is meant by “…calibrated form..” “…were seropravalence…”. Was this done by one or two authors? Why are the variables displayed in the tables not described in this part? |
| **'author response'** | Data extraction was conducted by three authors using a standardized form. |
| **'author action'** | This step is now clarified in the method section. Risk factors and symptoms have been added in the table. |
|  |  |
| **'reviewer comment'** | Results: In general: please provide a clear structure with subtitles if necessary (Study characteristics, prevalence by continent and over time, symptoms, risk factors, etc. |
| **'author response'** | Thank you for the advice for clarification |
| **'author action'** | The text has been amended accordingly. |
|  |  |
| **'reviewer comment'** | Figure 1: some inconsistencies with spacing’s before, after etc. I wonder why the duplicates were not removed earlier in the process, its written that removal was done after full-text analysis? The preprint articles from the reference list screening do not appear in the flow-diagram. |
| **'author response'** | Thank you for these relevant remarks. Duplicates were identified before and sometimes after retrieval when the title in medRxiv and published format in other databases differed. In the PRISMA flow-chart, full text reading comes afterwards. |
| **'author action'** | The figure has been fully revisited and updated |
|  |  |
| **'reviewer comment'** | Line 100: do you mean with OCDE? Is Iran an OECD country? I suggest order this part according to the geographical location (as in the Figure 2). |
| **'author response'** | Done |
| **'author action'** | We corrected the mistyping and ordered the presentation by location. |
|  |  |
| **'reviewer comment'** | Figure 2: please clearly indicate what is shown in this figure. Please add some description in the main text as well as in the Figure caption. It should be clear what comparison is shown here. Is it the lowest and the highest prevalence by continent? I think it would be much more helpful to show the % separated (all prevalence estimates) by continent and ordered by time. This would allow good comparisons. |
| **'author response'** | According to the new set of studies and increase of data collected, as well as to these remarks, the Figure 2 has revisited and presented in more clear strategy by time and location.  This is helpful for interpretation of the spread of the pandemic phenomenon. |
| **'author action'** | Figure 1 is now presenting point estimates over time in each continent (except Australia where only one estimate was available). |
|  |  |
| **'reviewer comment'** | Supplementary table: The items displayed do not match the variables planned to be extracted in the methods section. Please use the term sample instead of population (which is usually the target for the estimates). I strongly suggest to include the table in the manuscript rather than in the supplement. In any case, the table layout should be improved (format as references, repeat the titles for each page, exact dates or at least clearly declare time points of assessments). |
| **'author response'** | In the previous format, time points of assessments were displayed in an extreme right column, and may have not been visible. |
| **'author action'** | We have replaced the term population by the term sample where appropriate, rearranged the table format, and included the table in the manuscript as Table 1. |
|  |  |
| **'reviewer comment'** | The summary for seroprevalence should be consistent and self-explanatory. E.g. 9 of 904 instead of percent, first round, second round (what does it mean?) |
| **'author response'** | The summary of seroprevalence estimates have been now presented in a consistent way across all references. |
| **'author action'** | The Table 1 has been amended accordingly |
|  |  |
| **'reviewer comment'** | Line 113: I do not understand this sentence. What is Q1-Q3? Quartiles for the minimum? |
| **'author response'** | Q1 and Q3 are traditional mathematic expression for first and third quartiles. Adding % makes it more explicit. |
| **'author action'** | This sentence has been rewritten |
|  |  |
| **'reviewer comment'** | Line 125: I suggest moving this to the study characteristics section |
| **'author response'** | We agree with this suggestion |
| **'author action'** | Done |
|  |  |
| **'reviewer comment'** | Discussion Line 150: I do not understand this sentence |
| **'author response'** | This sentence has been deleted |
| **'author action'** | This sentence has been deleted |
|  |  |
| **'reviewer comment'** | Line 155: this should be stated more clearly already in the results in order to make this statement here. |
| **'author response'** | Only twelve articles explicitely assessed asymptomatic populations |
| **'author action'** | This is now specified in the result section |
|  |  |
| **'reviewer comment'** | Para 2: I think statements such as “Populations were not differently affected….” are not allowed based on the design of your study. Even if the majority of studies found no difference, this statement is not correct (if a meta-regression would have been conducted, some statements in this direction could have been made). Statements like most studies…found no…would be ok. |
| **'author response'** | Thank you for this comment. |
| **'author action'** | The text has been amended. |
