# Supplementary material for: Seroprevalence and SARS-CoV-2 invasion in general populations: A scoping review over the first year of the pandemic
Source: PLoS One. 2023 Apr 19;18(4):e0269104. doi: 10.1371/journal.pone.0269104 (PMC10118383; doi:10.1371/journal.pone.0269104)
Supplement: Attachment — Submitted filename: Response to Reviewers.docx [file pone.0269104.s003.docx]

|  | Reviewer #1 |
| --- | --- |
| **Requirement** | Thank you for addressing the suggested revisions. I know it must have taken a lot of work to include 71 additional studies so well done. |
| **'author response'** | We appreciate the nice comment, thank you |
| **'author action'** | -- |
| **Requirement** | Five minor comments:  Please add to the limitations that you did not conduct a grey literature search and the rationale for this |
| **'author response'** | We have added this to the limitations  . |
| **'author action'** | This is now added line 225-228:  Second, we did not include a grey literature search because we did not know how to search such literature in the particular context of this pandemic with so much suspicion around non-peer–reviewed publications. |
| **Requirement** | Page 13 line 273 – comment regarding blood donor studies “completely fading away in 2020.” They didn’t. Many blood donor studies are still ongoing. Please remove that statement or revise to indicate that they “decreased in frequency” |
| **'author response'** | We agree with this suggestion |
| **'author action'** | This sentence has been rewritten line 204 |
| **Requirement** | Page 15 line 328: “which peaked at 69% in England”. I would remove this part of the statement. This English study was likely of very poor quality and 69% is probably a very biased estimate. |
| **'author response'** | We did not assess the quality of the studies at at the time of study selection nor during extraction; this criterion was not part of our exclusion criteria. Other studies with lower prevalence may also be of poor quality and therefore biased. This is probably a point to mention in the limitation. We acknowledge that it is not relevant to mention it in the conclusion because it was not fully the scope of the review. |
| **'author action'** | As a limitation, we have added a statement warning the reader that we did not assess the methodological quality of the studies, lines 231-232, and removed this part of the statement from the conclusion. |
| **Requirement** | Page 15, Line 328-329 “As the geographical representation becomes broader with time, the representation of the spread of the pandemic in the world population improves.” I would remove this statement – I don’t understand what it means or what its purpose is. |
| **'author response'** | This sentence was not quite clear. |
| **'author action'** | This sentence has been deleted |
| **Requirement** | Although the manuscript is understandable overall, there are grammatical issues throughout. It would be useful to have two colleagues that have not seen this work proofread for grammar prior to final submission. PLOS One does not copy edit manuscripts and I do not have the time to type out all the errors for correction. I apologize for not having the time for this but it is the reality. |
| **'author response'** | We are sorry for the grammatical errors and we requested a native English-speaking medical editor to revise our manuscript. |
| **'author action'** | The article has been edited for English language. |
